# Development of Allele-Specific Therapeutic siRNA in Meesmann Epithelial Corneal Dystrophy

**DOI:** 10.1371/journal.pone.0028582

**Published:** 2011-12-12

**Authors:** Haihui Liao, Alan D. Irvine, Caroline J. MacEwen, Kathryn H. Weed, Louise Porter, Laura D. Corden, A. Bethany Gibson, Jonathan E. Moore, Frances J. D. Smith, W. H. Irwin McLean, C. B. Tara Moore

**Affiliations:** 1 Division of Molecular Medicine, Colleges of Life Sciences and Medicine, Dentistry & Nursing, University of Dundee, Dundee, Scotland; 2 Department of Paediatric Dermatology, Our Lady's Hospital for Sick Children, Crumlin, Dublin, Ireland; 3 Institute of Molecular Medicine, Trinity College Dublin, Dublin, Ireland; 4 Department of Ophthalmology, Ninewells Hospital & Medical School, Dundee, Scotland; 5 School of Biomedical Sciences, University of Ulster, Coleraine, Ireland, United Kingdom; University of Florida, United States of America

## Abstract

**Background:**

Meesmann epithelial corneal dystrophy (MECD) is an inherited eye disorder caused by dominant-negative mutations in either keratins K3 or K12, leading to mechanical fragility of the anterior corneal epithelium, the outermost covering of the eye. Typically, patients suffer from lifelong irritation of the eye and/or photophobia but rarely lose visual acuity; however, some individuals are severely affected, with corneal scarring requiring transplant surgery. At present no treatment exists which addresses the underlying pathology of corneal dystrophy. The aim of this study was to design and assess the efficacy and potency of an allele-specific siRNA approach as a future treatment for MECD.

**Methods and Findings:**

We studied a family with a consistently severe phenotype where all affected persons were shown to carry heterozygous missense mutation Leu132Pro in the KRT12 gene. Using a cell-culture assay of keratin filament formation, mutation Leu132Pro was shown to be significantly more disruptive than the most common mutation, Arg135Thr, which is associated with typical, mild MECD. A siRNA sequence walk identified a number of potent inhibitors for the mutant allele, which had no appreciable effect on wild-type K12. The most specific and potent inhibitors were shown to completely block mutant K12 protein expression with negligible effect on wild-type K12 or other closely related keratins. Cells transfected with wild-type K12-EGFP construct show a predominantly normal keratin filament formation with only 5% aggregate formation, while transfection with mutant K12-EGFP construct resulted in a significantly higher percentage of keratin aggregates (41.75%; p<0.001 with 95% confidence limits). The lead siRNA inhibitor significantly rescued the ability to form keratin filaments (74.75% of the cells contained normal keratin filaments; p<0.001 with 95% confidence limits).

**Conclusions:**

This study demonstrates that it is feasible to design highly potent siRNA against mutant alleles with single-nucleotide specificity for future treatment of MECD.

## Introduction

Meesmann epithelial corneal dystrophy (MECD) is a hereditary eye disorder that is inherited in an autosomal dominant manner [1], [2]. Previously, we demonstrated that the molecular basis of MECD is a dominant-negative mutation in either of the *KRT3* or *KRT12* genes encoding keratins K3 or K12, respectively, which are expressed only in the keratinocyte cells of the anterior corneal epithelium [3]. The K3/K12 intermediate filament cytoskeleton imparts mechanical strength to these keratinocytes and therefore, dysfunction of this system leads to mechanical fragility of the anterior corneal epithelium. Since then, more than 20 distinct disease-causing mutations have been reported in K3 and K12 in MECD families; see intermediate filament mutation database, http://www.interfil.org/ [4]. The vast majority of MECD mutations, like that of all the other keratin disorders, are point mutations leading to amino acid substitutions within the keratin rod domain. Such mutations are known to be highly detrimental to keratin function through a dominant-negative pathomechanism [5].

MECD can cause foreign body sensation and photophobia but is often asymptomatic and detected in the course of routine eye examination. Slit lamp observation shows multitudinous microcysts within the anterior epithelium, evident even in symptomless individuals. A subtle feature is the presence of gray serpiginous lines within the anterior epithelium. Rarely, a more severe phenotype with corneal erosions and scarring can lead to significant loss of visual acuity requiring treatment by keratoplasty or corneal grafting [6], [7], [8]. To date no molecular mechanism has emerged for the greater clinical severity observed in some families. Similarly, no therapy has been developed which addresses the underlying pathology associated with any of the corneal dystrophies.

In recent years, RNA interference (RNAi) has been identified as a highly potent and specific means of silencing genes in a user-defined manner. This process of sequence specific, post-transcriptional inhibition of gene expression has great potential to be developed as a novel therapeutic approach for a number of disorders where gene inhibition is predicted to be therapeutic [9]. As a dominant-negative disease, MECD represents an ideal model for RNAi therapy development aimed at specific ablation of the mutant allele. In particular, the small size, transparency and accessibility of the anterior corneal epithelium combine to make this tissue ideal for siRNA delivery via topical formulations. In addition, the microcysts that are the hallmark of the disease process can be readily imaged in a non-invasive manner to monitor efficacy *in vivo*. Although siRNA therapy of this type requires design of allele-specific silencing reagents for each individual mutation [10], [11], most MECD patients in Europe have a common founder mutation Arg135Thr [3], [12].

Here we assessed an extended family with MECD where the clinical presentation was consistently severe. Molecular genetic analysis, coupled with protein expression studies in cultured cells, pointed to a molecular mechanism for the unusually severe phenotype in the kindred. We used a K12-luciferase reporter gene assay to systematically perform a sequence walk across the point mutation in order to identify siRNAs that potently and specifically inactivated the mutant allele without any gene silencing effect on the normal allele. We also confirmed findings at a protein level and in a cell-based model system, where lead inhibitors significantly reversed the cytoskeletal aggregation phenotype.

Overall this therapeutic approach shows promise, and coupled with a suitable delivery system for the ocular surface, such reagents have huge potential for treatment of MECD, with the added potential of designing similar siRNAs for dominant-negative mutations in many other corneal dystrophies via a personalized medicine approach.

## Results

**Figure 1:**
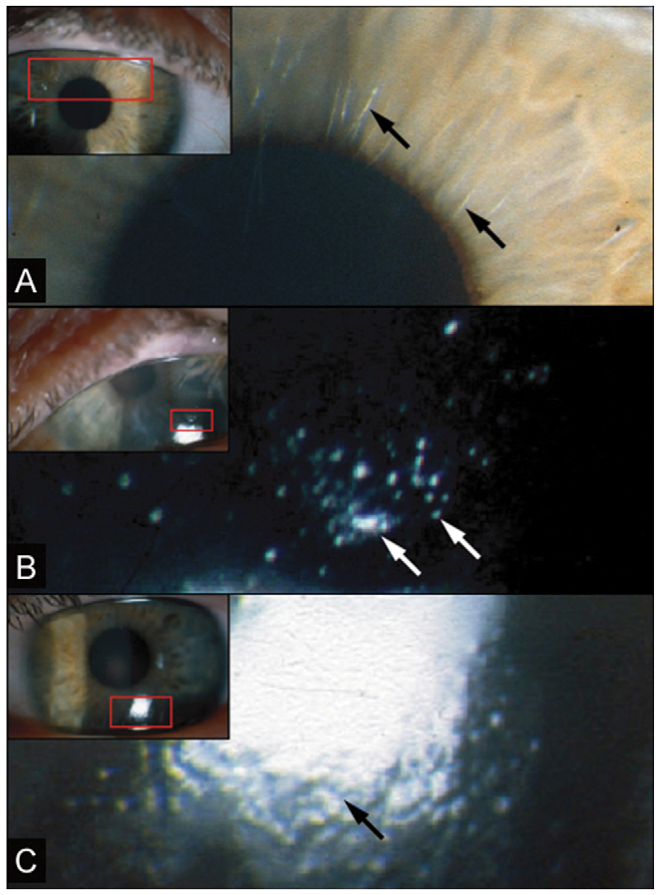
Clinical characteristics of MECD. (A) Ophthalmic examination of the proband from one of the families showed grey lines in the corneal epithelium, one of the characteristics of MECD. Slit lamp photography revealed multiple microcysts in the anterior epithelium (B) and uneven corneal topography secondary to damage and scarring of the underlying basement membrane (C).

An extended British multigenerational family (MECD Family 1) was studied showing autosomal dominant inheritance of MECD (Fig. S1). All members of the kindred exhibited an unusually severe clinical presentation (Fig. 1). Specifically, affected persons tended to have recurrent corneal erosions with scarring leading to pain and reduced visual acuity. Slit lamp photography revealed not only multiple microcysts in the anterior epithelium but also uneven corneal topography secondary to damage and scarring of the underlying basement membrane and anterior stroma (Fig. 1A–C). A number of individuals in the family had been treated with corneal grafting, which is uncommon in MECD.

**Figure 2:**
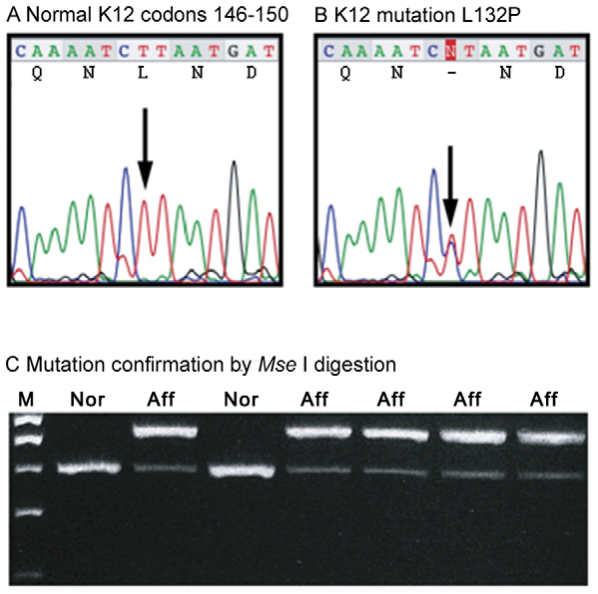
Molecular genetic analysis of K12 for the MECD families. (A) Sequence excerpt from K12 exon 1 derived from a normal person. (B) A novel heterozygous transition mutation was found in the proband: c.395T>C in exon 1 of the KRT12 gene predicting a leucine to proline amino acid substitution at codon 132 (designated p.Leu132Pro, or for brevity, Leu132Pro). (C) The mutation destroys a recognition site for the restriction enzyme *Mse* I site, which allowed rapid exclusion of this sequence change from 50 normal, unrelated and ethnically matched control individuals by *Mse* I digestion of KRT12 exon 1 PCR fragments. Nor = unaffected members of Family I; Aff = affected persons from Family 1. White arrowhead = filamentous keratin; blue arrowhead = keratin aggregates.

Hot spot mutation regions exons 1 and 7 of the *KRT3* gene, encoding protein K3, and exons 1 and 6 of the *KRT12* gene, encoding K12, were screened for mutations by direct DNA sequencing of specific PCR products. This revealed a novel heterozygous transition mutation in the proband and all affected members of Family 1, c.395T>C in exon 1 of the *KRT12* gene (Fig. 2A&B). This mutation predicts the amino acid change leucine to proline at codon 132 (Leu132Pro). This mutation is located in the functionally critical helix initiation motif at the start of the helix 1A domain of the K12 polypeptide. The mutation destroys a recognition site for the restriction enzyme *Mse* I, which allowed rapid exclusion of this sequence change from 50 normal, unrelated and ethnically matched control individuals by *Mse* I digestion of *KRT12* exon 1 PCR fragments as demonstrated in Fig. 2C. The c.395T>C mutation was also confirmed by PCR and DNA sequencing using a different primer set.

**Figure 3:**
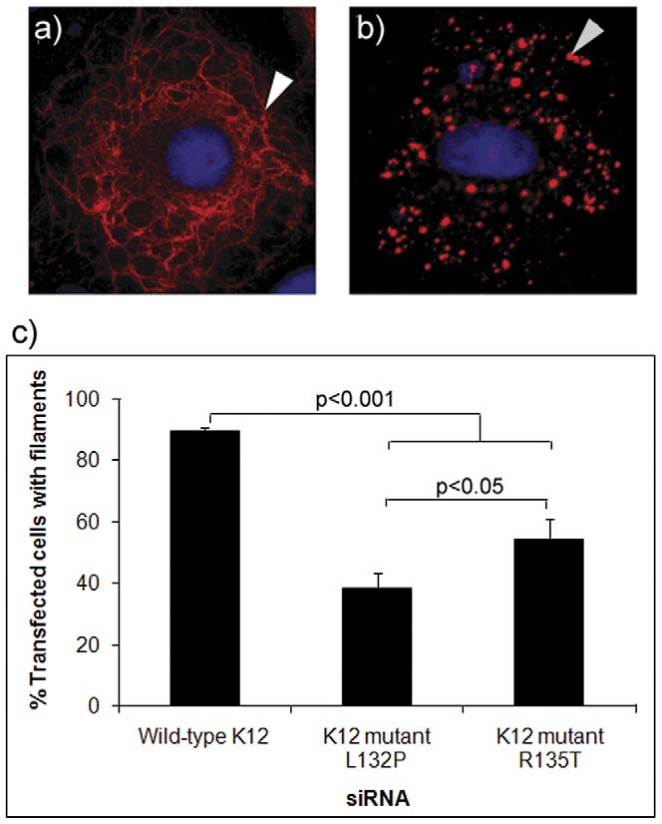
Aggregate formation in cells transfected with mutant keratins. (A) Co-transfection of both untagged wild-type K3 and K12 cDNA constructs into Ptk2 cells resulted in production of a well-developed intermediate filament network by 24–48 hours post-transfection as demonstrated by immunofluorescence staining (antibody AE5 against K3). (B) Co-transfection of mutant versions of K12, Leu132Pro or Arg135Thr, with wild-type K3, resulted in a high percentage of K3/K12-positive cells containing dense cytoplasmic aggregates of K3/K12 protein demonstrated by the red foci. Original magnification 60×. Scale bar = 10 µm. (C) The percentage of filamentous versus aggregative-containing cells was scored from 100 cells transiently transfected with wild-type or mutant forms of K12, at 24 hours post-transfection. Error bars represent standard error of the mean of replicate experiments. Wild-type K12 showed close to 100% filamentous keratin while both mutations showed a significant difference in the percentage of cells containing aggregates, when compared to wild-type (p<0.001). The common MECD mutation Arg135Thr showed significantly less aggregate formation compared to the Leu132Pro mutant (p<0.05).

The most common K12 mutation in Europeans associated with typical, mild MECD is Arg135Thr [3], [12]. To investigate why mutation Leu132Pro produced a more severe phenotype, both untagged and EGFP-tagged cDNA expression constructs were generated for wild-type human K3 and K12 to allow visualization of keratin filament or aggregate formation in transfected cells. Similarly, the expression constructs for the K12 mutants Leu132Pro and Arg135Thr were generated by site-directed mutagenesis. Initial attempts to express wild-type K12 by transient transfection into the simple epithelial cell line PtK2 [13], a standard assay of keratin filament function [14], [15], did not result in filamentous keratin staining but instead, dense aggregates were seen over a range of time points (not shown). PtK2 cells are used to assay keratin filament assembly because flat morphology and the presence of few endogenous keratins allow clear visualization of the cytoskeleton, compared to corneal or epidermal keratinocytes [15], [16]. This observation that transient expression of wild-type K12 did not result in filament formation suggested that type I keratin K12 is unable to form polymers with the endogenous keratins within PtK2 cells, namely K7, K8, K18 and K19 [16], and probably requires the presence of its natural type II keratin polymerization partner, K3. In contrast, other type I keratins, such as K14 or K16, form normal-appearing filaments in PtK2 cells [14], [15]. Subsequent co-transfection of both wild-type K3 and K12 constructs resulted in production of a well-developed intermediate filament network by 24–48 hours post-transfection (Fig. 3A). Co-transfection of mutant versions of K12, Leu132Pro or Arg135Thr, with wild-type K3, resulted in a high percentage of K3/K12-positive cells containing dense cytoplasmic aggregates of K3/K12 protein (Fig. 3B). Over-expression of wild-type keratins produces aggregates within the first 12 hours of transfection, however, these largely integrate into filament networks by 24–48 hours and persist in filamentous form at later time points [14], [15]. In contrast, dominant-negative mutant keratins produce aggregates that persist in a large percentage of cells at 24–48 hours post-transfection. By this means, a measure of the pathogenicity of particular mutations can be gained by measuring the percentage transfected cells showing filaments versus aggregates observed at, for example, 24 or 48 hours post-transfection [14], [15]. Thus, the percentage of cells showing filamentous K3/K12 staining at 48 hours was determined for wild-type, Leu132Pro and Arg135Thr versions of K12, co-transfected with K3 into PtK2 cells (Fig. 3C). Expression of K3 along with either of the K12 mutants resulted in a much lower percentage of filamentous keratin than K3 along with wild-type K12 and the difference was highly statistically significant for both Leu132Pro and Arg135Thr compared to wild-type (p<0.001). Importantly, Leu132Pro produced a consistently lower percentage of filamentous keratin than Arg135Thr (p<0.05; Fig. 3C), i.e. in this assay Leu132Pro is more disruptive to keratin function than Arg135Thr.

**Figure 4:**
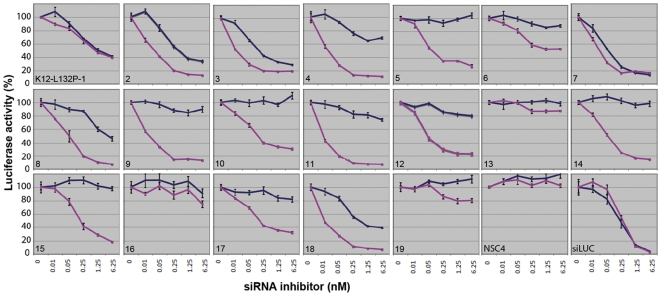
A comprehensive siRNA sequence walk for the K12 mutation Leu132Pro. This sequence walk shows the best siRNA was at position 9 (K12-L132P-9) which was very potent against the mutant reporter (pink line on plots) even at as low as 10 pM but did not significantly inhibit wild-type (blue line on plots) at concentrations as high as 6.25 nM. siRNA inhibitor K12-L132P-13 did not affect wild-type or mutant reporter while K12-L132P-2 and K12-L132P-3 knocked down both alleles to varying degrees NSC4 is a non-specific control siRNA, (negative control). siLUC is an siRNA specific for luciferase (positive control).

In order to develop a therapeutic starting point for MECD, a sequence walk was performed with all 19 possible siRNA inhibitors designed to be specific for the K12 Leu132Pro point mutation as demonstrated in Table S1. For each inhibitor investigated, the effect on K12-*firefly* luciferase reporter gene expression was determined in four replicate experiments over a range of siRNA molar concentrations (0, 0.01, 0.05, 0.25, 1.25 and 6.25 nM) against the wild-type and Leu132Pro mutant K12-*firefly* luciferase constructs. In each case, the data was normalized against co-transfected *Renilla* luciferase as a control for cell viability and transfection efficiency. Representative results are presented graphically in Fig. 4. A non-specific siRNA (NSC4) negative control had no inhibitory effect on either the wild-type K12 reporter or the Leu132Pro reporter, as predicted, while a known potent siRNA against *firefly* luciferase (siLuc), used as a positive control, knocked down the expression of both wild-type and mutant constructs roughly equally and over a wide range of siRNA concentrations (Fig. 4).

Of the mutant-specific siRNAs under test, some inhibited both wild-type and Leu132Pro mutant K12 equally, over a range of concentrations, such as K12-L132P-1, or K12-L132P-7 and therefore did not discriminate wild-type and mutant alleles. Some test inhibitors had little or no effect on expression of either the wild-type or mutant reporter gene expression, even at high concentrations, such as K12-L132P-13 or K12-L132P-16. However, from the 19 inhibitors, a number were able to specifically and potently inhibit the mutant allele with little or no effect on the wild-type allele, such as K12-L132P-4, 5, 9, 10, 11, 12, 14 and 15. Although all of these have good potential for therapeutic use, arguably the best inhibitor for further development was K12-L132P-9, where expression of the wild-type allele is not significantly knocked down, even at the highest concentrations, whereas the Leu132Pro mutant allele is potently knocked down even at the lowest siRNA concentration of 0.01 nM (Fig. 4).

**Figure 5:**
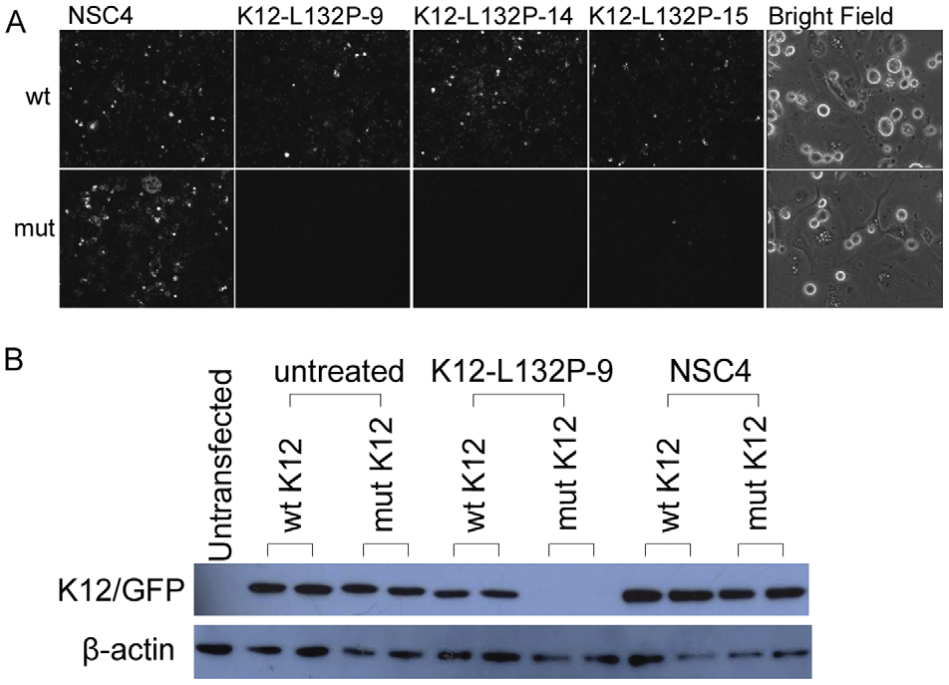
Differential inhibition of mutant K12 versus wild type by allele-specific siRNAs in cultured cells. (A) AD293 cells were transfected with wild-type or mutant K12-EGFP construct and siRNAs K12-L132P-9, 14 and 15 at a final concentration of 5 nM. Fluorescent imaging at 48 hours post-transfection showed that the expression of both wild type and mutant K12-EGFP constructs was almost equally strong for the cells treated with NSC4 while there was a significant reduction of fluorescence in the cells transfected with mutant K12-EGFP and inhibitors, although a subtle reduction of fluorescence in the cells treated with K12-L132P-9 or 15 was also observed. Original magnification 10× (except bright field image, 40×). Scale bar = 50 µm. (B) To confirm and quantify the highly differential inhibitory effect of K12-L132P-9 on mutant K12, western blot analysis was performed. AD293 cells were transiently co-transfected with either K12-L132P-EGFP expression construct and siRNA NSC4 (a non-specific control), or K12-L132P-9 at a final concentration of 5 nM. When cells were treated with K12-L132P-9, the mutant K12-EGFP was almost completely knocked down, while expression of wild type K12 construct treated with the same siRNA showed a negligible reduction compared with NSC4 treatment. Wt = wild type and mut = mutant.

To confirm the discriminative down-regulation effect of those siRNAs identified above, AD293 cells were transfected with wild-type or mutant K12-EGFP constructs and three representative discriminatory siRNAs K12-L132P-9, 14 and 15 at a final concentration of 5 nM. AD293 cells were used because they lack endogenous K3 or K12 expression and exhibit a high transfection efficiency. Non-specific siRNA NSC4 was used as a negative control and distilled water was used as an “untreated” control. Fluorescent imaging at 48 hours post-transfection showed that the expression of both wild type and mutant K12-EGFP constructs was almost equal for cells treated with negative control siRNA NSC4 (Fig. 5A). On the contrary, there was a significant reduction of fluorescence in the cells transfected with mutant K12-EGFP and inhibitors (Fig. 5A).

**Figure 6:**
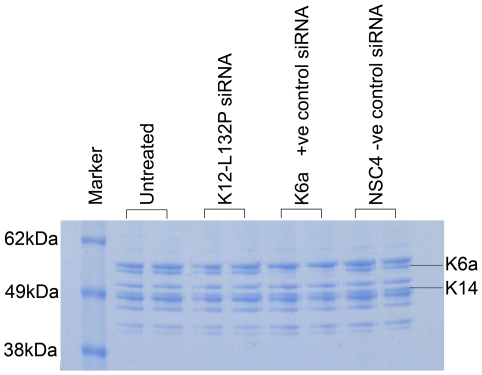
Lack of off-target silencing of other keratins by lead inhibitor for K12 Leu132Pro mutation, siRNA K12-L132P-9. SimplyBlue staining of cytoskeletal protein extracts from HaCaT cells revealed allele specific K12-L132P-9 siRNA had negligable effect on the range of endogenous keratins expressed in the HaCaT cell line. In contrast, a positive control siRNA directed against wild-type K6a demonstrated a dramatic decrease in K6a expression. Non-specific control siRNA NSC4 had no effect on keratin protein expression profile.

To confirm and quantify the highly differential inhibitory effect of the lead inhibitor K12-L132P-9 on mutant K12, western blot analysis was performed. Cells were transfected as described above with the expression of wild type and mutant K12-EGFP constructs similar in both untreated and NSC4 treated cells (equal loading was confirmed). In contrast, when the cells were treated with siRNA K12-L132P-9, mutant K12 protein expression was almost completely knocked out, although the expression of wild type K12 construct treated with the same siRNA showed a negligible reduction, comparable to the non-specific control NSC4 treatment (Fig. 5B). These data confirm that siRNA K12-L132P-9 is highly potent and highly specific for the K12 mutation Leu132Pro at the level of protein expression. The lead inhibitor (K12-L132P-9) was investigated for off-target effects on other keratins in HaCaT cells [17], which endogenously express a range of keratins, including K14, which is closely related to K12 in terms of sequence conservation. Coomassie blue stained gels showed no reduction on any keratins expressed in this keratinocyte cell line (Fig. 6). In contrast, a positive control siRNA against K6a [18], strongly knocked down K6a protein expression at this time point. Thus, this mutation-specific K12 siRNA appears to be highly specific.

**Figure 7:**
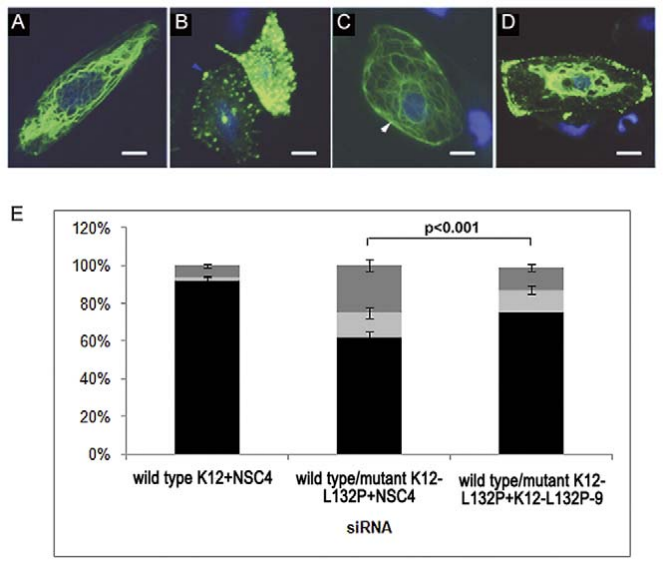
Therapeutic siRNA significantly reduces aggregate formation. Ptk2 cells were co-transfected with K3 and wild type K12-EGFP or mutant K12-EGFP or a 1∶1 ratio of both wild type and mutated K12-EGFP plasmids along with wild type K3 and mutant specific siRNAs and incubated for 72 hours. Filamentous keratin was visualized by direct fluorescence of EGFP-tagged K12. There was a significant difference in aggregate formation between cells treated with siRNA K12-L132P-9 and those treated with NSC4; p<0.001. Figure 7D is representative of a cell with correct filament formation while Figures 7B and 7D illustrate the formation of keratin aggregates. Within the graph, black = filament formation; light grey = occurrence of both filaments and aggregates; and dark grey = aggregates. Original magnification 60×. Scale bar = 10 µm, white arrowhead = filaments and dark arrowhead = aggregates.

Since primary epithelial cell cultures from affected patients were not available, a tissue culture model for the dominant-negative MECD disorder was established by co-transfecting PtK2 cells with a mixture of mutant and wild type K12 expression constructs along with wild type K3 expression construct. Similarly, cells co-transfected with wild-type K12-EGFP and K3 constructs were used as a model of normal corneal epithelia. The ability of lead siRNA inhibitor K12-L132P-9 to selectively block mutant K12 expression was assayed by counting the percentage of cells in which keratin filaments were in filamentous form or in aggregate form at 48 hours post-transfection in a blinded experiment. Cells transfected with wild type K12-EGFP construct predominantly formed normal keratin filaments (92.5%) with little evidence of aggregate formation (5%). In contrast, cells transfected with the mutant K12-EGFP construct contained a much higher percentage of keratin aggregates (41.75%; p<0.001), as shown in Fig. 7A–D. As expected for a dominant-negative mechanism, cells transfected with a mixture of wild-type and mutant (1∶1 molar ratio) K12 expression plasmids with wild-type K3 expression plasmids without siRNAs (data not shown) or with irrelevant NSC4 siRNA were defective in keratin filament formation (only 61.5% of the cells contained predominantly keratin filaments; p<0.001; Fig. 7E). Co-transfection of cells with a mixture of wild-type K12 and K3, and mutant K12, expression plasmids along with siRNA K12-L132P-9 significantly rescued the ability to form keratin filaments (74.75% of the cells contained normal keratin filaments; p<0.001; Fig. 7E).

## Discussion

In the dominantly inherited genetic disorders, where the pathomechanism is that of dominant-negative interference rather than loss-of-function/haploinsufficiency, therapeutic approaches such as gene replacement therapy are not appropriate and instead, a means of silencing mutant alleles would be a possible route to therapy [19], [20]. Over the years, a number of gene silencing methods have emerged, such as antisense RNA, morpholino oligonucleotides, or peptide nucleic acids, which can block translation of a target mRNA species of choice [10]. Unfortunately, because they are largely based on irreversible binding of the target mRNA by the antisense molecule, these systems are poorly discriminating for alleles that differ by only a few nucleotides or as the case is here, a single nucleotide. In recent years the use of synthetic siRNA, which exploit the naturally occurring RNAi pathway present within all human cells, has become a popular choice for development of therapy based on gene silencing [9], [21]. Within the cell, siRNA enters and forms the sequence scanning part of RNA-induced silencing complex (RISC) – a highly processive RNA-protein catalytic complex which functions by selecting and degrading target mRNA species [22]. siRNAs are typically 13 kDa, double-stranded 19-mer RNA molecules, flanked by single stranded 2-nucleotide overhangs [23] and are therefore much closer in size to a small molecule drug (typically <550 Da) rather than a gene therapy vector (typically 5–10 MDa). This has obvious advantages for delivery and currently, international efforts are on-going in both industry and academia to develop a non-invasive delivery vehicle for siRNA for skin [20] and cornea [24].

A major challenge to development of therapeutics aimed at silencing mutant alleles is the requirement for a high-degree of sequence specificity. In MECD and most of the other keratin disorders, the majority of causative mutations are point mutations [4], [25]. Remarkably, it has proven possible to develop potent, highly efficacious siRNA molecules that can discriminate a single nucleotide [21], [26], [27]. Here, we have adapted the recently reported fluorescent protein sequence walk methodology [27], to employ a luciferase co-reporter system. This allows for a higher throughput 96-well format assay, as well as internal normalization to control for cell viability and/or transfection efficiency. A standardized range of siRNA concentrations from 6.25 nM down to 10 pM, was used to measure potency, as well as sequence specificity. Some of the allele-specific inhibitors identified were highly potent with a high degree of single-nucleotide specificity, for example, siRNA K12-L132P-9 (Fig. 4), had a half maximal inhibitory concentration (IC_50_) of about 10 pM, which is better than many small molecule drugs in clinical practice. Remarkably, the best lead inhibitors developed here are capable of severely knocking down mutant keratin protein expression (Fig. 5), without any obvious off-target effects on other keratin proteins (Fig. 6) as well as reversing cytoskeletal dysfunction in a cell culture model system (Fig. 7). The inhibitors, which performed well in all three assays, are suitable for further preclinical development such as GMP manufacture and toxicology, to pave the way for future clinical applications.

Knock down of K12 mutant allele subsequent to application of allele specific siRNA therapy requires single copy expression of K12 to be sufficient for normal corneal epithelial function. Previous studies using gene targeted animal models, in which one allele of the murine *K12 gene (Krt1-12)* was ablated, show no corneal fragility phenotype [28]. Similarly, human subjects heterozygous for a K14 null mutation show no skin fragility phenotype [29]. These findings demonstrate there is no haploinsufficiency issue with certain type I keratin genes, including K12 and K14 and therefore one allele is sufficient to allow normal epithelial function, validating the mutation-specific siRNA approach taken here.

Although MECD is genetically heterogeneous, there are a number of recurrent mutations, including a common European founder mutation [3], [12], that are suitable for allele-specific approaches such as that taken here. Other types of corneal dystrophies arise from mutations in the transforming growth factor beta-induced gene (TGFBI, also known as BIGH3) on chromosome 5q31 [30], [31], [32]. The underlying pathology of both keratin and BIGH3 corneal dystrophies is very similar – both are predominantly caused by dominant-negative missense mutations. Thus, while MECD is ideal for therapy development and optimization, in the clinic BIGH3 may well be a major target, in addition to K3 and K12. A disadvantage of the BIGH3 dystrophies for siRNA development is that the extracellular aggregates found in these disorders take years or decades to develop clinically, making assays of efficacy difficult to model in cultured cells or animal models. The high turnover of the anterior epithelium in 5–7 days [33] coupled with the rapid appearance/disappearance of the intracellular mutant keratin aggregates in MECD combine to make this an ideal model corneal dystrophy for siRNA therapy. Lessons learned in development of specific inhibitors and their delivery to the cornea in MECD can then be rapidly translated to the more common and more clinically severe BIGH3 disorders.

The biggest hurdle to clinical application of these siRNAs is that of finding an efficacious and non-invasive delivery system. The cornea provides a highly attractive target tissue for siRNA therapy both in terms of optimization of delivery strategies and as a target tissue for corneal disease. As a target tissue, the corneal epithelium is a thin, four cell layer non-cornified stratified epithelium, and with no equivalent of the skin barrier layers to penetrate, delivery is easier than in epidermal field, where there is also a great deal of activity in siRNA therapy development. The cornea is therefore ideal as a proof-of-concept tool for translation of siRNA therapy into clinical practice as it is so easily accessible, disease status is easily monitored and the surface to be treated is small. Here we have demonstrated that it is possible to make highly potent and mutation-specific siRNA therapy reagents that target MECD-causing K12 mutations. Here, we chose the Leu132Pro mutation associated with a severe MECD phenotype, as there is great clinical need in the affected family. Similar siRNAs are in development for the other MECD associated mutations, including the more common Arg135Thr mutation.

The next step toward clinical application requires assessment of siRNA *in vivo* using knock-in reporter gene and phenotypic disease mouse models. Careful assessment of the specificity of the siRNA for the mutant allele at higher doses required for *in vivo* delivery ensuring significant suppression of the wild-type allele does not ensue, alongside studies of frequency and concentration of dosage requirements will greatly inform advances into clinical trials. The highly processive nature of RISC combined with the very potent nature of the Leu132Pro siRNA described here, should allow a relatively low dose of siRNA to be delivered to the cell. Coupled with a suitable delivery mechanism, be that chemical modification of the siRNA itself, formulations or enhancers to give optimal cell entry or physical methods to increase cell uptake, these small synthetic molecules have great potential for treating corneal dystrophies.

## Materials and Methods

### Subjects

An extended British family with MECD was studied where the clinical presentation was consistently severe. All individuals were examined using a slit lamp by an experienced ophthalmologist. EDTA blood samples were obtained from the patients with written informed consent and Institutional Ethics Committee approval was obtained that complied with the Declaration of Helsinki Principles (Tayside Committee on Medical Research Ethics A, study number 249/95). DNA was extracted following standard procedures. Molecular genetic analysis of keratin genes *KRT3* and *KRT12 w*as performed as previously described by Irvine et al 1997 [3].

### Expression constructs

Total human corneal epithelial mRNA was extracted from human corneal tissue using the QuickPrep micro mRNA purification kit (Amersham Biosciences UK Ltd., Bucks, UK). The full length K12 cDNA, including 3′UTR but excluding the poly-A signal was obtained by PCR using the following primers: K12M1F 5′-CCT TCC CCA GGC CAT GGA TCT and K12M1R2 5′-CAA TTA ACT CTA TTA AAA CAA. The full-length human K3 coding sequence was amplified using primer pair K3F1 5′-CTT CGC CAA GCT CCT TAC C and K3R1 5′-CGA TGC TGA TGC GTG CTC T. PCR fragments were cloned into pCR2.1 (Invitrogen, Paisley, UK) and fully sequenced. To allow EGFP (enhanced green fluorescent protein) tagging, the termination codons of the wild-type and Leu132Pro mutant K12 cDNAs were eliminated using QuickChange™ Site-Directed Mutagenesis (Stratagene; Agilent Technologies, Cheshire, UK) using primers 5′-ATT GAA GAA CTA ATG GGA AAT TTC ACA AGA TC and 5′-GAT CTT GTG AAA TTT CCC ATT AGT TCT TCA AT. To generate EGFP expression constructs, *EcoR* I – *Bgl* II fragments were subcloned into vector pEGFP-N3 (Clontech, France) *Eco*R I and *Bam*H I. These constructs were designated as K12-EGFP and K12Leu132Pro-EGFP. The unmodified K3 cDNA was subcloned into pcDNA3 for mammalian expression (using *EcoR* I).

### K12-Luc reporter constructs

Mutations Leu132Pro and Arg135Thr were introduced into the K12 cDNA clone (in pCR2.1) with the QuickChange™ Site-Directed Mutagenesis (Stratagene; Agilent Technologies, Cheshire, UK), using primer pairs 5′-ACT ATG CAA AAT CCT AAT GAT AGA TTA and 5′-TAA TCT ATC ATT AGG ATT TTG CAT AGT (for Leu132Pro), and 5′-AAT CTT AAT GAT ACA TTA GCT TCC TAC and 5′-GTA GGA AGC TAA TGT ATC ATT AAG ATT (for Arg135Thr). To generate luciferase reporter constructs for siRNA screening, wild-type and mutant K12 cDNA fragments were subcloned into psiTEST-LUC-target vector (York Bioscience Ltd, York, UK) using *Not* I and *BamH* I. A *Renilla* luciferase expression construct (pRL-CMV, Promega, Southampton, UK) was used as an internal control for both cell viability and transfection efficiency.

### siRNA design

siRNAs (19+2 format; 19-nucleotide duplex with two 3′ uracyl nucleotide overhangs) were synthesized by MWG Biotech AG (Ebersberg, Germany) to screen all possible target sequences containing the Leu132Pro mutation, plus positive and negative controls. This T>C point mutation occurs at position 395 in the K12 coding sequence, numbering by convention where the initiating ATG,  = 1 and excluding the 5′UTR (c.395T>C). The sense and antisense strands for the mutation-specific siRNA reagent designated as K12-L132P-1 were 5′-AGA AAC UAU GCA AAA UCC UU and 5′-GGA UUU UGC AUA GUU UCU UU, respectively (mutant position underlined). siRNAs K12-L132P-2 through K12-L132P-19 were designed similarly and the sense strands are represented in Table S1. The sense and antisense strands for positive control siRNA (siLuc) targeting the *firefly* luciferase gene were 5′-GUG CGU UGC UAG UAC CAA CUU and 5′-GUU GGU ACU AGC AAC GCA CUU, respectively. The sense and antisense sequences for the non-specific control siRNA (NSC4; an inverted bacterial β-galactosidase sequence) were 5′-UAG CGA CUA AAC ACA UCA AUU and 5′-UUG AUG UGU UUA GUC GCU AUU, respectively.

### Cell culture

Human AD293 embryonic kidney cells (Invitrogen, Paisley, UK) and *Potorous tridactylis* (rat kangaroo) kidney cell line PtK2 [34] were maintained in DMEM (Invitrogen, Paisley, UK) containing 4.5 g/L glucose and 10% fetal calf serum (FCS; Invitrogen, Paisley, UK), supplemented with 2 mM L-glutamine and 1 mM sodium pyruvate. Cells were incubated at 37°C with 5% CO_2_ supplement and passaged following standard laboratory procedures.

### Luciferase Reporter Assay

AD293 cells were cells of choice for the luciferase assay as they do not express endogenous K12 or K12 and therefore we can control what is expressed. Cells were plated at 7×10^3^ cells/well in a 96-well plate, resulting in 80% cell confluence at the time of transfection. Cells were transfected in quadruplicate with a mixture of 15 ng *firefly* luciferase expression plasmid, 1 ng *Renilla* luciferase expression plasmid and siRNA (final concentration 0.01–6.25 nM per transfection) which was diluted to 25 µl in optiMEM medium (Invitrogen, Paisley, UK). 1 µg Lipofectamine 2000 (Invitrogen, Paisley, UK) was incubated in optiMEM medium at room temperature for 5 minutes. This mixture was added to the nucleic acids and incubated for 20 minutes at room temperature, before addition to cells. The Dual-Luciferase Reporter Assay (Promega, Southampton, UK) was used to measure the effect of siRNA on luciferase expression 24 hours after transfection. The assay was used according to manufacturer's instructions. Briefly, the medium was removed and cells were washed with PBS before replacement with Passive Lysis Buffer (Promega, Southampton, UK). Cells were shaken on a plate shaker for 15 minutes to ensure cells were fully lysed, before the activities of both *Firefly* and *Renilla* luciferase were measured sequentially using the LUMIstar OPTIMA (BMG Labtech, Aylesbury, UK).

### Western blot analysis

Cells were incubated for 48 hours after transfection, then washed with PBS and lysed with NuPAGE 1× loading and solubilization buffer (Invitrogen, Paisley, UK). The extracted protein samples were denatured at 100°C for 5 minutes before being resolved on a 4–12% NuPAGE gel with SDS-MOPS running buffer (Invitrogen, Paisley, UK). To ensure adequate protein transfer and equal loading of samples the membrane was stained with Ponceau S (Sigma, Poole, UK) before immunoblotting. The K12 fusion protein tagged with EGFP (K12wt-EGFP and K12Leu132Pro-EGFP) was stained with anti-EGFP antibody (diluted 1∶1000; Roche, West Sussex, UK) for 1 hour at room temperature and anti-ß-actin antibody (diluted 1∶5000; Sigma, Poole, UK) was used as an endogenous control. Membranes were washed with TBS-Tween for 10 minutes three times before being stained with anti-mouse HRP secondary antibody (diluted 1∶1000; Dakocytomation, Cambridge, UK) for 1 hour at room temperature. After another three 10 minute washes with TBS-Tween, the membrane was treated with the mixture of ECL solution I and II (1∶1) for 2 minutes and X-ray film exposures were developed.

### Transient transfections of HaCaT cells

The lead inhibitor for K12 Leu132Pro mutation was investigated for off-target effects on other keratins in HaCaT cells; known to express a wide range of keratins. HaCaT cells were left untreated or incubated with the lead inhibitor for K12 Leu132Pro (K12-L132P-9), or a positive control siRNA against the 3′UTR region of K6a or a non-specific control siRNA (NSC4). Transfections were set up in duplicate in 12 well plates using Lipofectamine RNAiMax (Invitrogen, Paisley, UK) according to manufacturer's instructions for the ‘reverse transfection’ protocol. Briefly, 2 µl siRNA (5 µM) was diluted in 200 µl Opti-MEM medium, 2 µl Lipofectamine RNAiMax was added, mixed and incubated at room temperature for 20 minutes. 200 µl siRNA/Lipofectamine RNAiMax complexes were added to each well. Trypsinized HaCaT cells (8×10^4^ cells in 1.8 ml DMEM, 10% fetal bovine serum) were added to each well to give a final siRNA concentration of 5 nM. Cells were incubated at 37°C for 72 hours before cells were harvested for insoluble cytoskeletal protein extraction.

### Cytoskeletal extractions

Cytoskeletal extracts were made 72 hours post-transfection using a low salt, high salt buffer system as previously described [35]. Cytoskeletal protein samples were heated in the presence of reducing agent to 70°C for 10 minutes and resolved on 4–12% NuPage Bis Tris gels (Invitrogen) with SDS-MOPS running buffer (Invitrogen, Paisley, UK). A See Blue Prestained protein standard (Invitrogen) was used to allow accurate determination of protein size. Gels were stained with SimplyBlue SafeStain (Invitrogen) and each lane examined for the presence of cytokeratins based on known molecular weight.

### Keratin aggregation assay

Ptk2 cells were chosen for this assay as their flat morphology allows for easier imaging of aggregate or filament formation. Cells were plated at 1×10^5^ cells per well in a 24 well plate, each well contained a sterile 13 mm diameter cover-slip and cells were co-transfected in quadruplicate with wild-type, mutant, or a 1∶1 mixture of wild-type and mutant K12 expression plasmids along with a wild-type K3 expression construct (400 ng DNA per transfection in total) in 1 µg Lipofectamine 2000 and optiMEM medium (Invitrogen, Paisley, UK). In siRNA experiments, siRNA was co-transfected at a final concentration of 5 nM. Cells were incubated for 72 hours with a change of medium at 24 hours. After 72 hours, cells were fixed with 3% paraformaldehyde for 20 minutes. The keratin cytoskeleton was visualized either by immunoflurorescence staining (K3 antibody AE5; Abcam, Cambridge, UK) or directly via EGFP. Nuclei were counterstained with 4′,6-diamidino-2-phenylindole before being mounted onto slides with hydramount medium. At least 100 cells per coverslip were analyzed for the presence of filaments only, aggregates only or both filaments and aggregates. Statistical analysis was performed on aggregate cell counts using a one-way analysis of variance (ANOVA) with a p<0.05 considered significant. Statistical analysis was performed with the use of SPSS version 17.

## Supporting Information

Figure S1 **Figure demonstrates the pedigree of MECD family 1.** The arrow denotes the proband.
(TIF)

Table S1 **siRNA sequence walk against K12 mutation Leu132Pro (DNA mutation c.395T>C).** Bold denotes mutated nucleotide. The sequences of a non-specific control siRNA (NSC4) and a positive control against firefly luciferase (siLuc) are also shown.
(DOC)
