# Supplementary figures and images for: Development of Allele-Specific Therapeutic siRNA in Meesmann Epithelial Corneal Dystrophy
Source: PLoS One. 2011 Dec 12;6(12):e28582. doi: 10.1371/journal.pone.0028582 (PMC3236202; doi:10.1371/journal.pone.0028582)

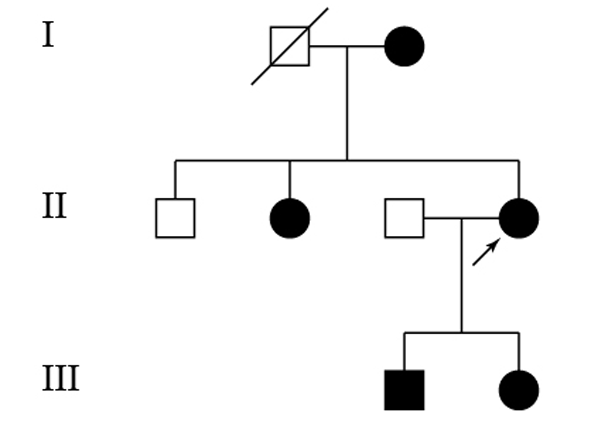

Supplement: Figure S1 — Figure demonstrates the pedigree of MECD family 1. The arrow denotes the proband. (TIF) [file pone.0028582.s001.tif]
