# Supplementary material for: Development of Allele-Specific Therapeutic siRNA in Meesmann Epithelial Corneal Dystrophy
Source: PLoS One. 2011 Dec 12;6(12):e28582. doi: 10.1371/journal.pone.0028582 (PMC3236202; doi:10.1371/journal.pone.0028582)
Supplement: Table S1 — siRNA sequence walk against K12 mutation Leu132Pro (DNA mutation c.395T>C). Bold denotes mutated nucleotide. The sequences of a non-specific control siRNA (NSC4) and a positive control against firefly luciferase (siLuc) are also shown. (DOC) [file pone.0028582.s002.doc]

| K12 | 5'-TCTGGATCAGAAAAAGAAACTATGCAAAATC**T**TAATGATAGATTAGCTTCCTACCTGGATAAG-3' |
| --- | --- |
| K12 L132P Mutant | 5'-TCTGGATCAGAAAAAGAAACTATGCAAAATC**C**TAATGATAGATTAGCTTCCTACCTGGATAAG-3' |
|  |  |
| K12-L132P-1 | AAGAAACUAUGCAAAAUC**C**UU |
| K12-L132P-2 | AGAAACUAUGCAAAAUC**C**UUU |
| K12-L132P-3 | GAAACUAUGCAAAAUC**C**UAUU |
| K12-L132P-4 | AAACUAUGCAAAAUC**C**UAAUU |
| K12-L132P-5 | AACUAUGCAAAAUC**C**UAAUUU |
| K12-L132P-6 | ACUAUGCAAAAUC**C**UAAUGUU |
| K12-L132P-7 | CUAUGCAAAAUC**C**UAAUGAUU |
| K12-L132P-8 | UAUGCAAAAUC**C**UAAUGAUUU |
| K12-L132P-9 | AUGCAAAAUC**C**UAAUGAUAUU |
| K12-L132P-10 | UGCAAAAUC**C**UAAUGAUAGUU |
| K12-L132P-11 | GCAAAAUC**C**UAAUGAUAGAUU |
| K12-L132P-12 | CAAAAUC**C**UAAUGAUAGAUUU |
| K12-L132P-13 | AAAAUC**C**UAAUGAUAGAUUUU |
| K12-L132P-14 | AAAUC**C**UAAUGAUAGAUUAUU |
| K12-L132P-15 | AAUC**C**UAAUGAUAGAUUAGUU |
| K12-L132P-16 | AUC**C**UAAUGAUAGAUUAGCUU |
| K12-L132P-17 | UC**C**UAAUGAUAGAUUAGCUUU |
| K12-L132P-18 | C**C**UAAUGAUAGAUUAGCUUUU |
| K12-L132P-19 | **C**UAAUGAUAGAUUAGCUUCUU |
|  |  |
| NSC4 | 5'-UAGCGACUAAACACAUCAAUU |
| siLuc | 5'-GUGCGUUGCUAGUACCAACUU |
